# The Emergence of *Clostridium difficile* Infection among Peripartum Women: A Case-Control Study of a *C. difficile* Outbreak on an Obstetrical Service

**DOI:** 10.1155/2011/267249

**Published:** 2011-07-24

**Authors:** Jennifer A. Unger, Estella Whimbey, Michael G. Gravett, David A. Eschenbach

**Affiliations:** ^1^Department of Obstetrics and Gynecology, University of Washington, Seattle, P.O. Box 356460, WA 98195-6460, USA; ^2^Department of Medicine, University of Washington Medical Center, Seattle, WA 98195-0001, USA

## Abstract

*Objective*. An outbreak of 20 peripartum *Clostridium difficile* infections (CDI) occurred on the obstetrical service at the University of Washington Medical Center (UWMC) between April 2006 and June 2007. In this report, we characterize the clinical manifestations, describe interventions that appeared to reduce CDI, and determine potential risk factors for peripartum CDI. *Methods*. An investigation was initiated after the first three peripartum CDI cases. Based on the findings, enhanced infection control measures and a modified antibiotic regimen were implemented. We conducted a case-control study of peripartum cases and unmatched controls. *Results*. During the outbreak, there was an overall incidence of 7.5 CDI cases per 1000 deliveries. Peripartum CDI infection compared to controls was significantly associated with cesarean delivery (70% versus 34%; *P* = 0.03 ), antibiotic use (95% versus 56%; *P* = 0.001), chorioamnionitis (35% versus 5%; *P* = 0.001), and the use of the combination of ampicillin, gentamicin, and clindamycin (50% versus 3%; *P* < 0.001 ). Use of combination antibiotics remained a significant independent risk factor for CDI in the multivariate analysis. *Conclusions*. The outbreak was reduced after the implementation of multiple infection control measures and modification of antibiotic use. However, sporadic CDI continued for 8 months after these measures slowed the outbreak. Peripartum women appear to be another population susceptible to CDI.

## 1. Introduction

The patient populations susceptible to *Clostridium difficile* infection (CDI) have now broadened to include pregnant women. In nonpregnant populations, both the incidence and severity of CDI have increased over the past decade. Recent large CDI outbreaks in Canada and U.S.A. demonstrated an increase CDI infection rate from a baseline of 2–6 infections per 1,000 hospitalized discharges (HD) in the 1990's [1–3] to 10–20 infections per 1,000 HD during recent outbreaks [4, 5]. As with nonpregnant patients, the incidence of CDI also has increased significantly in peripartum women. Using the Nationwide Inpatient Sample of all payer U.S. hospital discharges, the number of nationally reported peripartum CDI cases doubled from 129 cases in 1998 to 294 cases in 2006; the estimated CDI incidence among peripartum women increased significantly from about 0.4 to 0.7 per 100,000 deliveries over this period [6]. While the apparent lower rate of CDI in peripartum than nonpregnant patients explains the sporadic reporting of peripartum CDI [7–10], severe manifestations including septic shock, toxic mega colon, and even death occur in the peripartum population [8–10].

Antibiotics significantly decrease both maternal and neonatal infections, but they also are the primary risk factor for CDI, the leading cause of nosocomial infectious diarrhea [11]. Antibiotics disrupt normal bowel flora and promote colonic *C. difficile* overgrowth and subsequent exotoxin production. Prolonged antibiotic and multiple antibiotic uses are particularly associated with CDI [12]. Exposure to *C. difficile* spores occurs from direct transmission among hospitalized patients or indirectly through fomites and healthcare workers [1, 13]. Thus, CDI risk factors in nonpregnant populations also include prolonged hospitalization as well as underlying disease, ICU care and elderly age [2].

Up to 50% of pregnant women now are exposed to antibiotics during a hospital delivery; prophylactic antibiotics are used for the 30% of women undergoing cesarean section in the U.S [14] and for the 15–20% of women with vaginal Group B streptococcus colonization to prevent neonatal infection [15]. Additionally, about 10% of women develop chorioamnionitis or postpartum endometritis infection requiring antibiotics [16]. Although single extended spectrum antibiotics provide comparable infection cure rates to multiple antibiotic regimens for postpartum endometritis [17], gentamicin and clindamycin with or without the addition of ampicillin continue to be a popular regimen to treat peripartum infection [18].

The University of Washington Medical Center (UWMC) is a 450 bed tertiary care teaching hospital with a high-risk referral obstetrical service and 2200 annual deliveries. In April 2006, the first case of peripartum CDI in two years was identified; over the following fifteen months, a total of twenty peripartum CDI cases were documented. Only two peripartum CDI cases were identified at UWMC in the prior five years. In this report, we sought to (1) characterize the clinical manifestations and outcomes of the first reported sustained peripartum CDI outbreak, (2) outline specific infection control measures and antibiotic modifications that may have limited the outbreak, and (3) determine potential risk factors of peripartum CDI through a case-control study. 

## 2. Materials and Methods

Peripartum was defined as four weeks before and four weeks after delivery. A case of peripartum CDI was defined by diarrhea and evidence of CDI documented by either a positive assay for *C. difficile* A or B toxin in the stool or colonic histopathology characteristic of *C. difficile* infection in a peripartum female. The presence of toxigenic *C. difficile* was identified in fecal specimens assayed simultaneously for *C. difficile* common antigen and toxin A by enzyme immunoassay (Triage *C. difficile* Panel). Specimens that were antigen positive, but toxin A negative were cultured for *C. difficile*, followed by PCR molecular testing for *C. difficile* 16 S gene and toxin B gene (an internally validated UW assay). In addition, at the discretion of the primary care provider, fecal specimens were assayed for cytotoxin B demonstrated by cytotoxic effects on human diploid fibroblast cells that were neutralized by *C. difficile* antitoxin (an internally validated UW assay) [19]. Several cases had more than one stool sample submitted for diagnostic testing. In such cases, the data was verified to represent the test date that corresponded to the diagnosis of CDI, and results were consolidated in the result section and in Table 1.

### 2.1. “Bundle” Interventions

**Figure 1:**
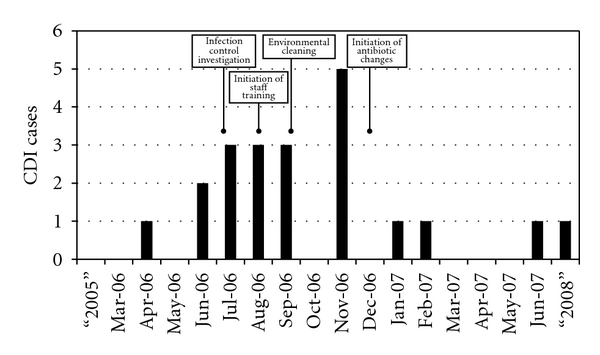
Clostridium difficile infection outbreak timeline and interventions.

After the first three cases of CDI, the UWMC Infection Control Department started an investigation and infection control audit July 2006 (Figure 1). By August 2006, step-wise comprehensive infection control measures similar to the previously described “bundle” approach [5] were initiated on the obstetrical unit (Table 3). 

Patients with confirmed CDI were placed in strict contact isolation that consisted of single room occupancy and gown and glove used by all visitors and personnel. All patients with diarrhea were placed on contact precautions until a negative toxin result was available. Patients with confirmed CDI were initially treated with a ten-day course of oral metronidazole. 

Immediately following the first cases, all providers and staff underwent intensive formal education and training on CDI prevention strategies (Table 3). All health care providers were required to use contact precautions and soap and water hand washing before and after any contact with* C. difficile* positive patients. Contact precautions included the use of gowns and gloves with any contact with a presumed or confirmed CDI case. Further, a water-based scrub was required for the first surgical case of the day instead of the previously used alcohol-based scrub. A thorough cleaning of the antepartum, labor and delivery, postpartum and outpatient clinic areas took place in September 2006. All patient environment and equipment was disinfected using a chlorine-based product (Bru-Clean TbC) rather than the routine hospital quaternary ammonium disinfectants as currently recommended [12]. A provider change room was installed by the operating room on labor and delivery to make clean scrubs readily accessible to providers after all deliveries. Carpet in the provider workrooms was replaced with hard wood laminated floors. Steps were made for the immediate diagnoses of CDI among patients with diarrhea.

Finally, a multidisciplinary team of providers and infection control specialists reviewed the most common microorganisms causing peripartum infection [20] and institutional susceptibility data to commonly used antibiotics. Antibiotic treatment was standardized for chorioamnionitis and postpartum endometritis to reduce the utilization of multiple antibiotics with a reported high resistance to *C. difficile*. As a result, ticarcillin/clavulanate (Timentin) was routinely used to treat chorioamnionitis and postpartum endometritis. Clindamycin was restricted to severe or unusual infection. Ampicillin and erythromycin use was continued for preterm premature rupture of membranes (PPROMs). 

The infection control “bundle” strategies were progressively implemented until after the outbreak peaked. The antibiotic transition strategies were gradually phased in and became routine by early 2007. Infection control remains heightened on the unit including soap and water washing, frequent changing of scrubs, strict contact precautions, and a narrow spectrum of antibiotic choices. 

### 2.2. Case-Control Study

Peripartum CDI risk factors may differ from those reported in CDI of nonpregnant adults. Thus, we performed a case-control study comparing peripartum CDI cases to randomly chosen unmatched controls who delivered during the outbreak period of April 2006 to June 2007. Using a random number table, four controls (*n* = 80) per case were selected from a hospital perinatal database of deliveries during the study period. Demographic, clinical, laboratory and outcome data were abstracted from medical records of both cases and controls. Data abstracted included CDI risk factors such as age, underlying disease, specific peripartum antimicrobial indication and use, length of hospitalization, and mode of delivery. No patient was excluded. 

### 2.3. Statistical Methods

We performed chi-square (*χ* ^2^) and Fischer's exact tests for univariate analysis of categorical variables and Mann-Whitney *U *tests for continuous variables. All tests were 2 tailed, and a *P* < .05 was considered statistically significant. We performed a logistic regression using CDI as the outcome. The mode of delivery, antibiotic use, and use of the antibiotic combination of clindamycin, gentamicin, and ampicillin were included in the model. Statistical analyses were performed with SPSS for Windows, version 12.0 (SPSS). The study was approved by the UWMC Human Subjects Committee no. 36114 under minimal risk criteria. 

## 3. Results

Twenty peripartum CDI cases were identified during the fifteen-month outbreak. A total of 2671 deliveries occurred over this time for an incidence of 7.5 CDI cases per 1,000 deliveries. CDI was diagnosed by the presence of a positive* C. difficile *EIA assay for Toxin A alone in eight patients, by the presence of a positive *C. difficile *PCR and/or cytotoxin assay for Toxin B alone in six patients, and by the presence of positive assays for both Toxin A and B in six cases (Table 1). Three cases developed CDI during a separate antepartum admission, seven cases were diagnosed during their delivery hospitalization, and ten postpartum cases were diagnosed after hospital discharge, eight of whom required readmission. Hospital readmission occurred back into the postpartum ward.

No significant clinical or antibiotic management change occurred on the obstetrical ward prior to the outbreak. The first two cases of the outbreak were diagnosed during the antepartum period. The first patient received clindamycin alone for preterm labor GBS prophylaxis at 32 weeks gestation and developed diarrhea 7 days later. A positive cytotoxin assay for *C. difficile* Toxin B was identified in her stool on hospital day 8. The patient delivered during a second hospitalization at 36 weeks gestation (Table 1). The second case presented with PPROM at 27 weeks and received ampicillin, then amoxicillin, and erythromycin and other antibiotics (Table 1) for one week. CDI symptoms developed six days after discontinuing antibiotics. A positive EIA assay for *C. difficile *Toxin A was detected in her stool the next day. She remained hospitalized and delivered at 29 weeks gestation. 

Patients received antibiotics for both prophylaxis and to treat infections. The indication for antibiotic use and actual antibiotic exposures are presented in Table 1. Ten of the 20 cases received a combination of ampicillin, gentamicin, and clindamycin for chorioamnionitis or postpartum endometritis. Two additional cases received clindamycin: one alone for GBS prophylaxis and one together with other antimicrobials for mastitis. Thus, 12 of the 20 cases received a regimen that included clindamycin. Two cases received a cephalosporin alone for Cesarean prophylaxis. One patient diagnosed with CDI in September received no antibiotics. 

Antibiotics were used to treat chorioamnionitis and/or postpartum endometritis in 12 of the 20 patients. Three cases received antibiotics for infections unrelated to pregnancy; they were among the 5 patients with long-term antepartum hospitalizations for significant chronic illnesses including Marfan's syndrome, class RF diabetes, chronic hypertension, osteosarcoma, and sickle cell anemia with crisis. All of these 5 also had significant obstetrical complications, including premature delivery. 

A total of 8 patients required hospital readmission after delivery for diarrhea and fever. The readmission to the postpartum unit could have contributed to the outbreak from *C. difficile* contamination of this hospital area. Two discharged postpartum patients were treated with outpatient therapy. Extra days of hospitalization for those inpatients diagnosed with CDI cannot be precisely calculated, but the eight patients readmitted for CDI required a total of 22 extra inpatient days or an average of almost 3 extra days per case. 

The morbidity among the cases was significant. All 20 patients presented with diarrhea. Fever during CDI was documented in 16 cases, a leukocytosis of greater than 15,000 in 9 cases and a creatinine of greater than 1.0 in 4 cases. One patient developed septic shock and toxic mega colon, but no deaths occurred in this series. 

A 52-year-old postpartum case suffered a toxic megacolon and required an emergent colectomy despite prompt oral metronidazole treatment for one day and subsequent oral and rectal vancomycin. The patient had a twin gestation; one twin delivered vaginally and the second twin by emergent cesarean section. She received cefazolin prophylaxis with surgery. On postoperative day 2, she developed a 38.9° temperature and received ampicillin, gentamicin, and clindamycin for postpartum endometritis. Antibiotics were discontinued on the 4th postoperative day, but she developed diarrhea later that day. A positive EIA assay for *C. difficile* Toxin A and a positive cytotoxin assay for Toxin B were identified in her stool on postoperative day 5, and she was promptly placed on metronidazole. The next morning, she developed septic shock: oliguria, tachycardia, hypotension, a leucocytosis (12.9 THOU/*μ*L), and a creatinine of 2.4 mg/dL. Vancomycin was begun both by a nasogastric tube and rectally, and she was transferred to the intensive care unit. The patient's clinical condition worsened on postoperative day 7, and a total colectomy was performed. Histopathologic examination of the resected colon confirmed the diagnosis of toxin megacolon and pseudomembranous colitis. This patient had no significant baseline risk factors for CDI except for her age. 

All infection control measures were implemented by the end of January 2007 (Figure 1). Changes to antibiotic prescribing protocols were in place by spring of 2007. One additional cases of peripartum CDI was diagnosed in the 36 months since outbreak ended June 2007. This patient was not included in the report because she transferred with CDI from an outside hospital. She presented with sepsis, pseudomembranous colitis, and a positive Toxin B assay. 

### 3.1. Case-Control Study

Univariate analyses results comparing the 20 CDI cases with 80 randomly chosen unmatched controls are presented in Table 2. The two groups did not differ in age or ethnicity. Women with CDI were more likely than controls to have undergone a cesarean section (70% versus 34%; *P* = 0.03), been previously hospitalized during the pregnancy (55% versus 2.5%; *P* = 0.001), and have significant underlying illness (25% versus 7.5%; *P* = 0.04). Underlying illness in the case group included Marfan's syndrome, class RF diabetes, chronic hypertension, sickle cell anemia with sickle crisis, osteosarcoma, and a significant neck venous malformation.

CDI cases undergoing cesarean section often had a long labor and a diagnosis of chorioamnionitis or postpartum endometritis. CDI was associated with both chorioamnionitis (OR 10.2, 95% CI 2.9–39.9) and postpartum endometritis (OR 13.0, 95% CI 2.3–73.4). The use of any antibiotic was strongly associated with CDI (OR 14.8, 95% CI 1.9–115.8) as was the use of the combination ampicillin/gentamicin/clindamycin (OR 39.0, 95% CI 7.5–204.0). The combination of ampicillin/gentamicin/clindamycin was used for 10 cases and only 1 control (*P* < .001). Three or more intravenous antibiotic doses (range 3 to 40 doses) were received by 18 cases and only 14 controls (*P* < .001). 

Three risk factors in the univariate analyses were both strongly associated with CDI and with each other: cesarean section delivery, any antibiotic use, and use of ampicillin/gentamicin/clindamycin. Thus, these three risk factors were examined in a logistic regression. In a binary logistic regression model, the use of the combination ampicillin/gentamicin/clindamycin persisted as an independent risk factor for CDI (*P* < 0.001). This confirmed the strong association present in the univariate analysis. 

## 4. Discussion

This peripartum CDI outbreak is the largest sustained outbreak reported to date on a labor and delivery unit [6, 8–10, 21]. A PubMed search of English citations from 1966 to April 2011 confirms previous reports of only up to 4 peripartum CDI cases in one institution [9]. Prior reports estimated the rates of peripartum CDI to range widely from 0.4 to 0.7 per 100,000 deliveries where the diagnosis was made from national coding data [6] to 0.7 per 1000 admissions where the diagnosis was extracted from microbiology laboratory log data [21]. The case rate of 7.5 CDI infections per 1000 deliveries over the 15 months of this outbreak was comparable to the rate of 10–20 CDI infections per 1000 hospital discharges during recent CDI outbreaks in nonpregnant adults [4, 5]. In contrast to these outbreaks, our patients were young women without typical risk factors such as prolonged hospitalization, prior ICU stay, or, for many, significant underlying illness. 

Two well-recognized CDI risk factors were present in almost all but one case: prior antimicrobial use and hospital exposure. Antibiotic use is particularly high in modern delivery services; 56% of the UWMC control patients in this study received at least one antibiotic dose. Multiple antibiotics were used simultaneously and/or sequentially in 85% of the CDI cases, and 3 or more antibiotic doses were given to 90% of cases. 

Examination of antibiotics used prior to the development of CDI suggests that the regimen of ampicillin, gentamicin, and clindamycin was a major factor in the outbreak. Repeated doses of this antibiotic regimen were strongly associated to CDI in both the univariate and in the multivariate analyses, independent of the mode of delivery and any antibiotic use. This potent combination of antimicrobials is popular in labor and delivery units because of its wide microbial coverage [17, 18], and it was used at UWMC for both chorioamnionitis and postpartum endometritis for the past 20 years. Both the multiple antibiotic combinations and its long time of use at UWMC may have acted synergistically to contribute to the outbreak. 

*C difficile* recovered from cases was not tested for clindamycin resistance in our report, but antibiotic resistance contributed to hypervirulent CDI strains in other outbreaks [22]. Previous reports found that up to 80% of *C. difficile* isolates were resistant to clindamycin, [23] and 60% of our cases received clindamycin. Since the outbreak, Timentin^R^ has been used to treat chorioamnionitis and endometritis. The lack of Timentin^R^ (ticarcillin/clavulanate) resistance to *C. difficile* at UWMC and an expected comparable infection treatment result [17] made the switch logical. Timentin^R^ caries a <1% resistance to *C. difficile,* although extended penicillins, like all antibiotics, have been implicated in CDI.

A meta-analysis on the CDI risk from various antimicrobials listed, in order of higher to a lower magnitude includes: second and third generation cephalosporins, amoxicillin or ampicillin with clavulanic acid, antipseudomonal penicillins, clindamycin, quinolones, aminoglycosides, ampicillin, and penicillin [24]. However, in the recent serious hypervirulent NAP1/027 CDI Quebec outbreak, quinolones were particularly singled out with a population attributable fraction of 36% [25]. The NAP1 *C. difficile* strain is not only fluoroquinolone resistant, but the hypervirulence is derived from its ability to produce 16 to 23 times more toxin A and B in vitro than toxin type O strains [26, 27]. Fluoroquinolones are relatively contraindicated in pregnancy, so they were not a factor in this peripartum CDI outbreak. The 56% antibiotic use rate in a delivery unit such as at UWMC is both astounding and common. Obstetrical units should pay close attention to antibiotic use and be prepared to institute antibiotic rotation, such as occurred here. 

Strong environmental control measures and an infection control “bundle” together with antimicrobial stewardship are recommended to control a CDI outbreak [5, 21, 28, 29]. It is impossible to assess the relative impact of the environmental interventions compared to antibiotic change in the cessation of this outbreak. Stepwise environmental and behavioral infection control measures and antibiotic changes were put in place simultaneously until the outbreak ended. However, despite a marked reduction in CDI after these measures were in place in December 2006, sporadic CDI cases continued for 6 months. The infection control methods used for the UWMC outbreak included: education, hand washing with soap and water, attention to clean scrub clothes, gown and glove use, environmental chlorine-based cleaning of patient rooms and bathrooms, and all nursing and physician work areas, together with a change of antimicrobials. It should be noted that spores are more poorly inhibited by the newer alcohol-based water-free hand-washing solution than with soap and water, and therefore the mechanical removing of potential *C. difficile* spores by hand washing is recommended [12]. Still, the contribution to this outbreak of water-free-hand washing solution is unknown. Environmental cleaning with chlorine-based cleaning agents is effective to reduce CDI outbreaks [6, 12]. Though we could not determine which specific infection control measures ended the outbreak, only one case was reported since June 2007, and this patient was transferred with CDI from another hospital.

CDI is treated by discontinuation of the implicated antimicrobial and the administration of oral metronidazole for mild-moderate disease or vancomycin for moderately severe or persistent disease [12]. Oral metronidazole initially was used to treat all young UWMC peripartum women; it is inexpensive and therapeutically equivalent to vancomycin in clinical trials of moderate disease [28]. However, vancomycin is recommended for severe diseases, because it is not absorbed from the gut, it stops toxin production, its colon lumen concentration is 50 to 200 times higher than the MIC of *C. difficile, *and vancomycin resistant *C. difficile* has not been reported [30]. The severe CDI case resulting in a colectomy in this outbreak was treated for one day with metronidazole, as she appeared to have mild-moderate disease. She rapidly developed septic shock so that even immediate vancomycin therapy may not have influenced this malignant course of CDI. Other peripartum patients in published reports also required colectomy [9, 10]. Thus, obstetric providers need to be aware that peripartum patients with CDI are at risk for severe and rapidly progressing disease, possibly because of depressed immunity during pregnancy [9]. Moreover, women with underlying disease may be at increased risk of peripartum CDI. 

Hospital obstetrical units pose a unique opportunity for infection control. The environment is purposely designed to create intimacy and a “natural” environment for labor and delivery. However, as we found, this environment also provides ample reservoirs and transmission modes for *C. difficile* infections. Providers have frequent contact with fecal contents during a delivery and travel from one patient room to another in the labor and delivery unit. In these high volume units, rooms are quickly cleaned for reuse, and infectious disease recognition can be neglected. In this unit, readmission of postpartum patients to the postpartum ward also may have contributed to the outbreak. Great vigilance must be taken on multiple levels to decrease the exposure of patients to antibiotic harm and to vigorously work to identify and prevent outbreaks of CDI. 

## Figures and Tables

**Table 1:** Clinical characteristics and diagnostics of peripartum CDI cases.

Age	Parity	Gestational age at CDI onset (wks)	Significant underlying illness	Obstetric complications	Gestational age at delivery (wks)	Mode of delivery	Antibiotic type	Toxin A (EIA)	Toxin B gene (PCR)	Toxin B (cytotoxin)	Outcome (fever, diarrhea)	Hospital days due to CDI
31	0	31	Marfan's Synd.	PTL	36	VD	Clinda	NEG	ND	POS	F, D	Long LOS
39	1	27		Twins, PPROM, chorio	29	VD	Amp/Amox, Aug/Azy/Ery	POS	ND	ND	F, D	Long LOS
52	5	pp		Twins, PTL, PPEM	36	CS	Clinda, Amp/Gent/Clinda, Ceph	POS	ND	POS	F, D, Total Colectomy	8
19	1	pp		Chorio	41	CS	Amp/Gent/Clinda	POS	ND	POS	F, D	1 (Re-ad)
31	1	pp		PPEM	39	CS	Amp/Gent/ Clinda, Aug/Azy/Ery, Ceph	POS	ND	ND	F, D	0 (OP)
49	1	pp		Mastitis	42	CS	Clinda, Ceph, Aug/Azy/Ery, Other	POS	ND	ND	F, D	3 (Re-ad)
26	6	pp		PPROM, PPEM	34	VD	Amp/Gent/Clinda, Aug/Azy/Ery, Amp/Amox	POS	POS	POS	F, D	3 (Re-ad)
25	3	PP		Chorio, PP hysterectomy	37	VD	Amp/Gent/Clinda, Ceph	POS	ND	POS	F, D	3 (Re-ad)
23	1	PP		Chorio	39	CS	Amp/Gent/Clinda	NEG	POS	ND	F, D	1 (Re-ad)
24	1	pp	Neck venous malformation		37	CS	Ceph	NEG	POS	ND	D	0 (OP)
26	1	22	Class RF DM, CHTN	Fetal anomalies	23	VD	None	NEG	POS	ND	D	5
41	3	pp		Twins, Pulm. Embolus, PPEM	37	CS	Amp/Gent/Clinda, Aug/Azy/Ery, Ceph, Other	POS	ND	ND	F, D	Long LOS
21	1	26		PPROM, chorio	27	CS	Amp/Gent/Clinda, Aug/Azy/Ery, Ceph, Other	POS	ND	ND	F, D	0 (Ante)
25	1	pp		Chorio	41	CS	Amp/Gent/Clinda	POS	ND	POS	F, D	0 (OP)
29	3	pp			38	CS	Ceph	POS	ND	ND	D	0 (OP)
38	4	pp		Chorio	38	CS	Amp/Gent/Clinda, Ceph	POS	POS	NEG	F, D	4 (Re-ad)
20	1	pp	Osteosarcoma	Pyelo, severe GHTN	28	VD	Other	NEG	POS	ND	F, D	5 (Re-ad)
32	3	26	Sickle Cell Dis.	Pneumonia	36	CS	Aug/Azy/Ery, Other	NEG	POS	ND	F, D	Long LOS
22	1	pp		PPEM	41	CS	Ceph, other	POS	ND	ND	F, D	2 (Re-ad)
38	7	pp		Placenta previa, abruption, pyelo	30	CS	Other	POS	ND	ND	D	0 (OP)

(Obstetric complications: PTL: preterm labor, PPROM: preterm, premature rupture of membranes, chorio: chorioamnionitis, PPEM: postpartum endometritis, pyelo: pyelonephritis, GHTN: gestational hypertension, mode of delivery: VD: vaginal delivery, CS: cesarean section; antibiotic type: clinda: clindamycin, Amp/Amox: ampicillin or amoxicillin, Aug/Azy/Ery: augmentin, azythromycin, or erythromycin, Amp/Gent/Clinda: ampicillin, gentamicin, and clindamycin, Ceph: cephalosporin; test results: ND: not done; hospital days: LOS: length of stay, Re-ad: patient readmitted, OP: outpatient therapy).

**Table 2:** Selected characteristics of CDI cases and of randomly selected controls.

	Cases (*N* = 20)	Controls (*N* = 80)	OR (95% CI)	*P*-value
*Mean age ± S.D.*	30.6 ± 9.3	29.0 ± 7.2		0.2

*Ethnicity*				
Caucasian	12 (60%)	41 (51%)		
African American	3 (15%)	8 (10%)		
Hispanic	3 (15%)	14 (18%)		0.9
Asian/Pacific Islander	1 (5%)	8 (10%)		
Other/unknown	1 (5%)	8 (10%)		

*Mode of delivery*	14 (70%)	27 (34%)	4.6 (1.6–13.3)	0.03
Cesarean section				

*Significant underlying illness *	5 (25%)	6 (8%)	4.1 (1.2–15.2)	0.04

*Prior antepartum hospitalizations*	11 (55%)	2 (3%)	47.7 (9.1–250.0)	0.001

*Complications *				
Preterm labor	2 (10%)	8 (10%)	1.0 (.20–5.1)	1.0
PPROM	3 (15%)	4 (5%)	3.4 (.06–1.5)	0.1
Chorioamnionitis	7 (35%)	4 (5%)	10.2 (2.9–39.9)	.001
Postpartum endometritis	5 (25%)	2 (3%)	13.0 (2.3–73.4)	.003

*Any antibiotic use*	19 (95%)	45 (56%)	14.8 (1.9–115.8)	0.001

*Antibiotic combinations*				
Amp/Gent/Clinda	10 (50%)	2 (3%)	39.0 (7.5–204.0)	<.001
Clindamycin alone	2 (10%)	2 (3%)	4.3 (.57–32.9)	0.2
Cefazolin/Keflex	10 (50%)	24 (30%)	2.3 (0.9–6.3)	0.9
Other	6 (30%)	2 (3%)	16.7 (3.1–91.3)	.001

*>3 doses of IV antibiotics *	18 (90%)	14 (18%)	42.4 (8.8–204.0)	<.001

*Categorical variables testing using chi-square or Fischer's exact test; continuous variables tested using the Mann Whitney *U* test.

**Table 3:** Infection control measures used for an obstetrical service outbreak of *Clostridium difficile *Infection (CDI).

(1) *Contact precautions *	
(a) Intensive education and training in the fundamentals of infection control.	
(b) Contact precautions for all suspected and documented CDI cases.	

(2) *Hygiene *	
(a) Thorough hand hygiene with soap and water rather than an alcohol-based hand gel when caring for patients with suspected or documented CDI.	
(b) Water-based surgical scrub for the first case of the day, and when hands are visibly soiled.	

(3) *Positive protective equipment (PPE) for potential exposure *	
(a) Gowns and gloves for contact with any suspected and documented CDI cases.	
(b) Frequent change of scrubs and protective garments.	

(4) *Environmental and equipment cleaning *	
(a) Extensive environmental cleaning and disinfection of the entire unit and outpatient clinic with a hypochlorite-based disinfectant.	
(b) Replace carpet in provider work rooms with laminated hard wood floors	

(5) *Diagnosis and treatment *	
(a) Prompt diagnosis of patients with diarrhea.	
(b) Prompt treatment of documented CDI or suspected CDI in seriously ill patients.	
(c) Good antibiotic stewardship with minimal clindamycin and multiple antibiotic regimen use.	
